# Towards elimination of Lymphatic Filariasis in Kenya: improving advocacy, communication and social mobilization activities for mass drug administration, a qualitative study

**DOI:** 10.1186/s40794-022-00172-8

**Published:** 2022-06-06

**Authors:** Lydiah W. Kibe, Bridget W. Kimani, Collins Okoyo, Wyckliff P. Omondi, Hadley M. Sultani, Doris W. Njomo

**Affiliations:** 1 Kenya Medical Research Institute - Eastern & Southern Africa Centre of International Parasite Control (ESACIPAC), Nairobi, Kenya; 2 Ministry of Health, Kenya – Division of Vector-Borne and Neglected Tropical Diseases, Nairobi, Kenya

**Keywords:** Advocacy Communication and Social Mobilization, Lymphatic Filariasis, Mass Drug Administration, Neglected Tropical Diseases, Kenya

## Abstract

**Introduction:**

The Kenya Breaking Transmission Strategy for Neglected Tropical Diseases (NTD) from 2019 to 2023 intensifies advocacy, coordination, and partnerships. The purpose of this study was to explore views and experiences of stakeholders and health workers on ways of improving the Advocacy, Communication and Social Mobilization (ACSM) activities of Mass Drug Administration (MDA) for Lymphatic Filariasis (LF) programs through participatory approaches in Kilifi County, Kenya.

**Methods:**

Two wards were purposely selected in the Kaloleni sub-county, Kilifi County, where there was an average treatment coverage of 56% in 2015, 50.5% in 2016. Qualitative data collection methods were employed, which included participatory meetings with county stakeholders to understand their views, experiences, and suggestions on how ACSM strategies can be improved in MDA for LF. Twelve In-Depth Interviews (IDIs) were conducted (six with opinion leaders and six with Community Health Extension Workers (CHEWs) and two semi-structured interviews (SSIs) were held with county and sub-county coordinators involved in MDA administration. The aim was to better to understand their perceptions of the NTD program about ACSM, challenges to ACSM strategies, and ways to improve the strategies for ACSM in MDA for LF. The Data was organized and classified into codes and themes using QSR NVIVO version 12.

**Results:**

The study observed the low participation of stakeholders in the ACSM activities of MDA for LF and identified potential areas for stakeholders’ involvement to strengthen the activities. Challenges hindering effective implementation of ACSM activities include late delivery of Information, Educational and Communication (IEC) and few IEC materials, insufficient funding, inadequate time allocated to reach the assigned households with messages, messaging, and packaging of information for dissemination due to the vastness of the area. The stakeholders recommended innovative strategies and techniques to improve ACSM activities.

**Discussion and conclusion:**

The results of this study show key challenges to ACSM implementation of MDA for LF. Implementers need to pay attention to these challenges to enhance the effectiveness of MDA per the Kenya NTD Breaking Transmission Strategy. ACSM efforts in MDA for LF control and elimination should be linked with overarching efforts to mainstream partnerships and coordination in control and elimination.

**Supplementary Information:**

The online version contains supplementary material available at 10.1186/s40794-022-00172-8.

## Background

Lymphatic Filariasis (LF) has been recognized as a global public health problem affecting close to a billion people living in low-resource settings [1]. Sub-Saharan Africa (SSA) reports a substantial proportion of this burden, thus resulting in huge economic losses and disability due to the diseases [2–4]. To eliminate LF, the World Health Organization (WHO) recommends implementation of mass drug administration (MDA) in endemic countries, for a period of at least 5 years, with consistent high drug coverage levels above 65% of the population at risk [5].

In Kenya, LF is endemic in all the six counties of the coastal region along the Indian Ocean from Lamu County in the north to Kwale County in the south bordering northern Tanzania where ecological and entomological factors are suitable for its transmission [6]. The Kenyan Ministry of Health (MoH) launched the LF elimination program in 2002 in the then Kilifi District*^1^ and later scaled it up in Malindi and Kwale Districts in 2003. In 2011, Tana River and Lamu counties were included while Taita Taveta and Mombasa counties were included in 2015 and 2016 respectively to achieve a 100% geographical coverage [7]. Following LF transmission assessment surveys in 2015, a recommendation was made to have additional MDA in counties of Kilifi, Kwale and Lamu as Circulating Filarial Antigen (CFA) showed an overall prevalence of between 1.3% and < 1.7% [7]. The MDA implementation campaigns for 2016 - 2019 were not interrupted by budgetary, technical, and administrative challenges as was the case in the previous campaigns [8].

MDA for LF program is under the Division of Vector-Borne and Neglected Tropical Diseases, (DVB-NTD) in the Kenya Ministry of Health. The implementation is guided by the Kenya NTD breaking transmission strategy 2019-2023. The plan is to expand MDA coverage, implement Water, Sanitation, and Hygiene (WASH) interventions and implement Behavior Change Communication (BCC) as a comprehensive package [9]. MDA implementation is done through the County and Sub-county health departments for a period between three and 5 days every year. Community Drug Distributors (CDDs) administer to all persons aged 2 years and above, an annual dosage of diethylcarbamazine citrate (6 mg/kg) and albendazole (400 mg) through the door to door strategy. They also conduct sensitization and awareness creation activities, through the provision of information, education, and communication materials to community members. The materials which include posters, pamphlets, and banners are provided by the National NTD program through county NTD coordinators. Implementation is funded by donors through the National NTD program team which supervises the activities. From 2016 - to 2019 the MDA for LF program reported national drug coverage levels of 70%.

Given the multiple challenges to achieving high treatment coverage in community-based programs, it is important to identify and evaluate specific strategies and techniques that may have utility in increasing or sustaining high coverage. A systematic review by Silumbwe et., al has summarized the factors that shape the implementation of MDA for LF in sub-Saharan Africa [10]. One key highlight in the review was the creation of partnerships and collaborations which was essential in sustained and continued implementation, especially in ACSM activities. On the other hand, studies have found out that a lack of partnerships and collaboration and a top-down approach limits the involvement of partners and stakeholders [11].

Previous studies have reported challenges that negatively influence access to MDA for LF. Some of these studies highlight challenges such as; low knowledge of LF transmission, myths, and misconceptions about the drugs – drugs are for family planning, fear of side effects, low perception of risk, the timing of the campaigns [12] which can be addressed through effective ACSM activities. This study explored the views and experiences of stakeholders and partners on ACSM for MDA in the LF program. The result of this study will help improve ACSM activities through the participation of communities and other stakeholders during preparation, development, and dissemination. Consequently, this would help increase MDA access and reduce misconceptions through targeted strategies and techniques that are effective and culturally sensitive ACSM activities as a component of LF elimination in Kilifi County, Kenya.

## Methods

### Research design

This qualitative study used a mix of qualitative methods including stakeholders’ analysis using Venn diagrams, in-depth interviews with opinion leaders and health workers, and semi-structured interviews with NTD coordinators at the county and sub-county level. The purpose was to explore the views and experiences of different players in ACSM activities for MDA in the LF program and make recommendations for improvement.

### Study setting

**Fig. 1:**
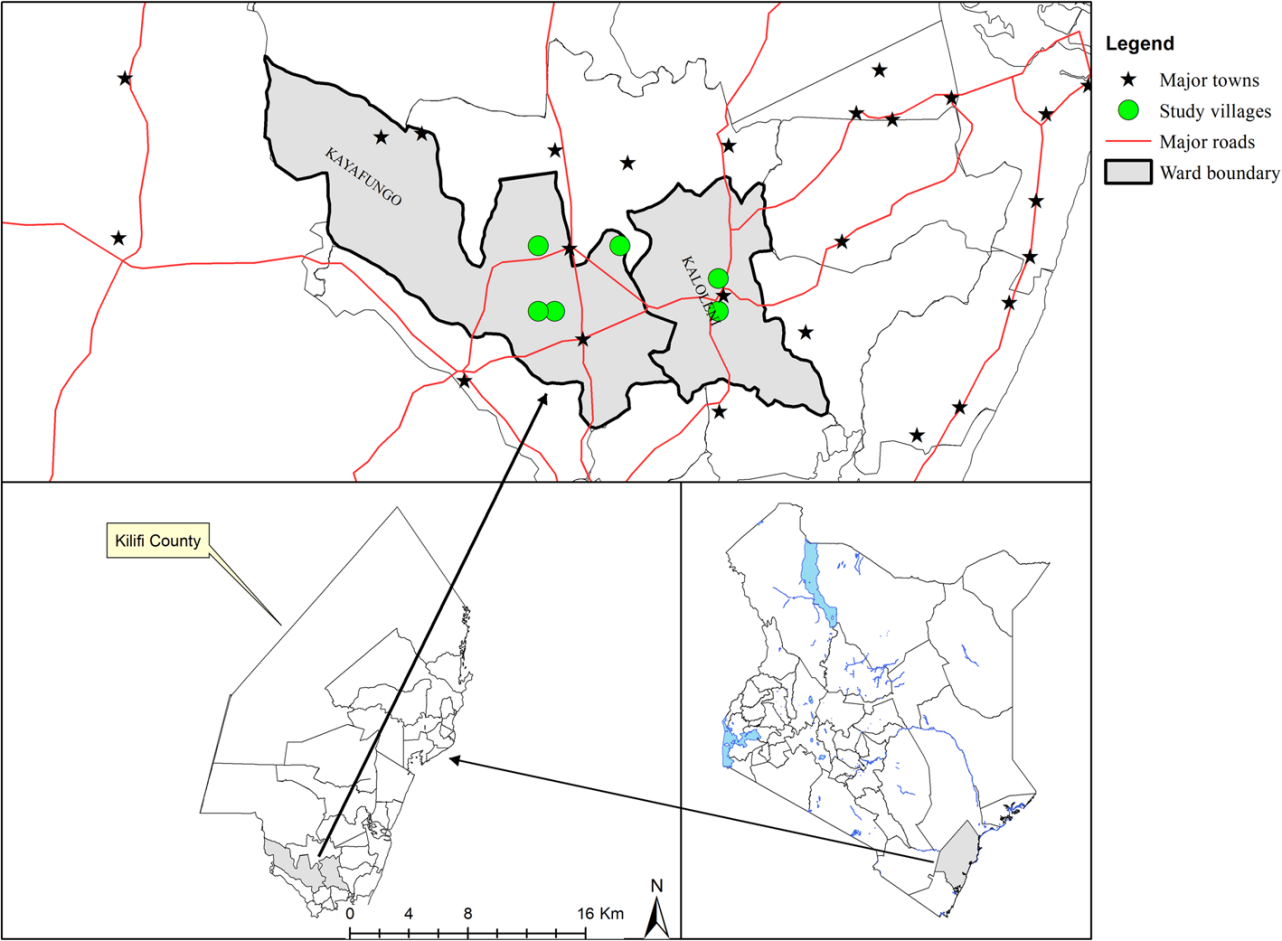
Map of the study area

Kilifi County has a population of 1,109,735 and covers an area of 12,245.90 km^2^ [8]. The County is located north and northeast of Mombasa, the second largest town in Kenya [13]. Administratively, the County is made up of 7 sub-counties: Kilifi North, Kilifi South, Kaloleni, Rabai, Ganze, Malindi, and Magarini. The County is endemic for LF caused by *Wuchereria bancrofti* and has a prevalence of filarial antigenaemia of < 1.7% and a mean microfilarial density of < 25 MF/ml [7]. The current study was conducted in Kaloleni Sub - County which has a population of 159, 739 [8]. Two wards i.e. Kaloleni which has a population of 41, 689 of which an average of 36.8% is urban [8], and Kayafungo with a population of 22, 250 people of different ethnic backgrounds formed the study area (Fig. 1: Map of the study area). The residents mainly belong to the Mijikenda ethnic group, which is a Bantu group comprising of nine sub-groups with a majority (45%) being Giriama. The main form of livelihood within this community is subsistence farming. Based on the 2015 and 2016 MDA Programme data, Kaloleni and Kayafungo wards were selected purposively for the study. In 2015, Kaloleni ward achieved average treatment coverage of 58% and Kayafungo, 54%, and in 2016, Kaloleni 62% and Kayafungo 39% all below the recommended minimum treatment coverage of 65% (MOH, Kenya,)

### Data collection procedures

#### Focus Group Discussions with Community members

A total of Eight Focus Group Discussions (FGDs) were held with community members. They included adult and youth male and female single-sex groups to assess their perceptions related to ACSM ad activities and suggestions for improvement (Additional file 1: FGD guide txt). FGDs were held in a community venue convenient to the participants. An FGD comprised of between 10 and 12 participants and discussions lasted I hour. Discussions were moderated by experienced social scientists who were assisted by trained field assistants.

#### Stakeholders analysis on their participation in ACSM activities

Two meetings were held - one with the county and one with sub-county level personnel at the county and the sub-county level. The meetings aimed to share the objectives of the study and receive participants’ suggestions on how future MDA for the LF program could be improved. The NTD coordinators at the county and sub-county invited participants using pre-determined criteria. The criteria included: participation in previous MDA activities, resident of the County or Sub-County, the person interested in MDA, and must be community representatives. At the county level, the meeting was attended by twenty-nine participants who included County Health Management Teams (CHMT), partners representing religious groups, Non- Governmental organizations, Governor’s office representative, media houses, education and other government ministries, provincial administration, and youth and women groups’ representatives. The one-day meeting was officially opened by the County Health Minister and moderated by the County NTD coordinator. Minutes of the meeting were taken during the meeting. The Sub county-level meeting was attended by twenty-two participants who included Sub-County Health Management Teams (SCHMT), CHEWs, health facilities in charge, partners representing religious groups, non - Governmental organizations, area chief, youth, and business community. The one-day meeting was officially opened by the Sub County Health Officer and moderated by the Sub-County NTD coordinator. Minutes of the meeting were taken during the meeting (Additional file 2 and Additional file 3 on minutes of stakeholders meeting in Kilifi and Kaloleni respectively). The discussions revolved around the identification of partners, their participation in the past MDA for LF activities, and what kind of participation they could offer during MDA for LF activities. Venn diagram, a visualization, and participatory tool were used with the participants to determine the closeness of various stakeholders/partners to MDA for LF activities and their level of involvement in ACSM activities. The further a stakeholder or partner was from the center of the circle the lower the level of involvement. The size of the circle depicted their perceived importance in ACSM activities. All the stakeholders and partners identified were perceived as very important in the ACSM activities of MDA for LF.

#### In-depth interviews with opinion leaders and community health extension workers

A total of Twelve In-depth Interviews (IDIs) were conducted; six with Opinion Leaders (OL) and six Health Workers (HW). OL comprised of chiefs, village elders, business owners, teachers while health workers comprised of Public Health Officers, CHEWs, Nurses, Community Health workers. The purpose was to better understand their perceptions of the NTD program about ACSM activities, challenges to successful ACSM implementation, social mobilization strategies, and ways to improve ACSM for MDA. The interviews were expected to help shed light on strategies and ways of improving ACSM strategies for improving access to MDA activities in the area. The IDI participants were selected using predetermined criteria. The criteria for OL selection included the following: a respected and resourceful person in the community, a group leader, resident of the study area, consented to be interviewed while selection for HW included a CHEW from the study area; and willing to consent to be interviewed. Interviews were held until a point of saturation. The IDIs were tape-recorded with permission from all participants.

#### Semi-structured interviews

Two semi-structured interviews were conducted with NTD coordinators at the County and sub-county levels to generate information on previous processes of MDA and preferred processes, barriers affecting access to information and awareness creation, and existing opportunities for improved ACSM activities.

### Data quality and control

To ensure data quality and optimization, research assistants were recruited from the community, from four levels of education and above. They received a week-long training covering qualitative data collection methods, ethical procedures, interviewing, note-taking, transcription, and translation. Interview guides were translated from English to Kiswahili and Kiswahili to English to reconcile discrepancies and differences. Field assistants were trained and supervised by experienced qualitative social science researchers. All issues emanating from interviewing and data capture were discussed with the research assistants and resolved in the field. After interviews field assistants reviewed their notes and added to any gaps observed. All recordings were coded and uploaded to the computer. The recordings were transcribed into English and typed in Ms. Word at the end of the interview sessions. The researchers supervised the transcription process to ensure accuracy and consistency between recording and transcription.

### Data processing and analysis

Audio recordings of the FGDs, IDIs were transcribed immediately following the discussions, and then translated from Swahili to English as appropriate. Transcripts were assigned unique identifiers such as FGD-KLN-CM-AW-003; IDI-KYF-OL-002; SSI_NTDC_01 etc. Field notes were incorporated in the written transcripts as additional data. The semi-structured interviews were typed and saved in MS word. All documents from FGDs, SSIs, IDIs, and minutes of meetings were reviewed in detail then imported to Nvivo 12 Plus software [14] for further processing and analysis. Deductive analysis was used to categorize codes based on the study objectives. Data coding was done simultaneously with data collection to ensure that thematic saturation was monitored. Also, to ensure a fair interpretation of the data, the transcripts were initially coded by two researchers independently. The coding process involved a critical review of each transcript to identify emerging themes from the data. The two coders then met to compare their independently-identified themes during data coding. They revolved divergence by re-reading the relevant sections of the transcripts together and agreed on the best fit interpretation of the data. The major and sub-themes are discussed below, supported by relevant quotes from the transcripts. Preliminary findings of the study were presented to the stakeholders at the county level to help improve the MDA activities. Quotes from participants were used to support the themes.

## Results

The results are presented with the relevant verbatim quotes according to the four thematic areas that emerged from the data. These four thematic areas are summarised in Table 1. They were challenges affecting the effective implementation of ACSM activities of MDA for LF; Potential of stakeholders’ involvement and participation in resource mobilization for MDA delivery with a focus on ACSM activities and; the need for innovative strategies and techniques to improve ACSM preparation, development, and dissemination and finally challenges with morbidity management and disability prevention services. Whilst data were collected across various participant categories, no major differences in the discussions were noted. Each of the themes is discussed below.

**Table 1:** Themes, challenges and opportunities identified

Themes	Challenges	Opportunities
1. Potential of stakeholder’s involvement and participation in resource mobilization for MDA delivery with a focus on ACSM activities,	i. Low involvement of stakeholders and partners in MDA for LF ii. Communication or lack of communication on MDA for LF to stakeholders iii.	- Inform to stakeholders early - Plan the MDA for LF with stakeholders - Share roles
2. The need for innovative approaches and strategies to improve ACSM preparation, development and dissemination and	i. Insufficient knowledge on the transmission cycle thus perpetuating myths and misconceptions ii. The IEC materials are few, not in the native language and they are delivered late	- Enhanced Community meetings (Barazas) with awareness creation by health officers - Mobile phones using WhatsApp and radio programmes
3. Challenges affecting effective implementation of ACSM activities in MDA for LF	i. Individual decision not to take drugs ii. Pressure to meet set targets iii. MDA Planning, implementation and follow-up	- Increase awareness creation using innovative strategies - Increase the MDA time from 3 days to five days - MDA for LF awareness should be continuous
4. Challenges with morbidity management and disability prevention services.	i. Patients fear surgery thus refuse their names to be recorded ii. Insufficient facilities such as functional theatre to execute the surgeries and wards to admit patients after surgeries iii. Insufficient personnel to perform surgeries	- County governments to expand and equip theatres - Patients to be encouraged to have the operations

### Challenges affecting effective implementation of ACSM activities of MDA for LF

Stakeholders discussed the progress made by the Ministry of Health Kilifi County towards ACSM activities of MDA for LF elimination. The stakeholders shared life experiences. It was agreed that the county had made good progress noting that there was a significant increase in knowledge on LF over the last decade, since 2002 when the program was launched in the county. It was noted that despite efforts made by the national NTD division in soliciting funds and materials to support ACSM activities, the level of awareness was still low. The stakeholders discussed and identified the following reasons for low awareness. This included inadequate information on the transmission cycle which lead to myths and misconceptions, individual decisions not to take drugs, pressure to meet targets, and vastness of the area compounded by poor terrain and houses being far apart. Nonetheless, CDDs who are expected to walk to the assigned household were not able to reach out to all the households with MDA messages. Failure to address these issues could affect the uptake of drugs by the target communities. Each of these issues is elaborated below with selected quotes.

***Insufficient knowledge on the transmission cycle thus perpetuating myths and misconceptions*** Participants in IDIs and stakeholders’ meetings reported that inadequate knowledge on the LF transmission cycle and drugs administered during MDA for LF had perpetuated myths and misconceptions. Below are some extracts from participants:

*… information is not adequate; simply because, mostly we call people in meetings for awareness creation in social halls, but not all people come to these meetings and forums, maybe they are employed somewhere or maybe are struggling for their daily bread, so they do not come to the places where awareness is done, so that is the problem* (IDI-KLN-OL-003)

*… LF is a silent disease. You only see the manifestation. The symptoms are not related to LF e.g. fever. The community is not aware. For us to convince them to take drugs, some do not relate it with the transmission cycle. They relate it to witchcraft (*SSI_NTDC_01)

*… several people in the community still say the drugs are for family planning. And family planning you know it's a choice, so people were saying you were told to do family planning you refused that is why the government has come with these drugs so that when you take them you will not bear children anyone. Men will not be able to produce children. Now, those things came up because they didn't get enough awareness, they were just told these drugs are for swollen limbs and swollen genitals so they thought they were being told that so that they could take them (IDI-KYF-OL-001)*

#### Individual decision not to take drugs

Several issues were cited as influencing individual decisions to comply with taking the drugs. Participants in IDIs indicated that some residents would accept to be left with the drugs and promise to take them later which in most cases they did not. Reasons cited included literacy levels, low perceived benefits of the drugs, perceived beliefs and attitudes towards the drugs. Other reasons included making one sleepy, being afraid of the drugs, they make them feel hungry, the taste remains in the mouth for 2 days, they are very strong, the tablets are too many, those who have swollen libs think that the drug will heal them and they get disappointed when they do not see any change, they make one feel weak and fatigue. Below are selected comments from the participants interviewed:

*… during mobilization and sensitization, they will assure you that they will have understood and that they will take the drugs but refuse to take the drugs during the MDA day. Others will receive the drugs but fail to consume in your presence saying they will take the drugs after a meal and you know you will not be there to witness and confirm they have taken. It is hard because you know you cannot force an adult person or even know their intention* (IDI-KLN-HW-002*)*

*… .. there was one person I asked, despite the rest taking the drugs, why are you not willing he replied that because he does not know what the drugs are for, then he could not accept but promised to take them next time* (IDI-KLN-OL-004)

*… . many people are illiterate, they have their own beliefs concerning the drugs* (IDI-KYF_HW_001)

#### Pressure to meet set targets

The CDDs were assigned to every 500 households to provide awareness sessions before and during the MDA campaign. The MDA exercise takes 5 days (2 days’ awareness and 3 days for drug administration. Opinion leaders who sometimes accompany CDDs during the campaign confessed that CDDs lack time to create awareness and to persuade residents to take drugs owing to the high target during the campaign and vastness of the area.

*… if you probe for reasons why a person does not want to take drugs, you will not cover the assigned households … You see such people if you probe they say it’s just within themselves. And if you probe more we delay and we shall not cover the assigned households* (IDI-KLN-OL-004)

*The information given to the household is not enough because the speed the CDDs had was only to enter a household and give drugs according to the instruction there was not enough time to talk and explain more about the drugs to make the household owners understand. They were in a hurry to finish the target of the day* (IDI-KLN-OL-005)

*… It was like it did not reach all the people .... the area is so vast. If you have visited Kayafungo, the area is so vast therefore it did not reach everyone* (IDI-KYF-HW-001)

#### The IEC materials are few, not in the native language and they are delivered late

The IEC materials are important aids in creating awareness for MDA campaigns. During the interviews with county and sub-county NTD coordinators and CHEWs, it was reported that the materials were delivered late, were few, and were usually in Swahili and English instead of the native language, Giriama. Additionally, the materials are delivered during the time drugs are delivered to the counties a few days before the drug distribution exercise which gave CHEWs and CDDs limited time to conduct awareness and sensitization sessions. Examples of extracts from the transcripts are given below.

*We receive the materials late. They are delivered to the county and we should organize to collect them. Sometimes, they do not arrive at the sub-county* (SSI-SCNTD-01)

*The main challenge is that those IEC materials arrive late. They are delivered at the county headquarters a week before the exercise and they must be transported to the sub-county before they are distributed to CDDs* (IDI-KLN-HW-001)

*Posters are good because when the community see pictures they understand, but the problem is posters are always very few like we receive 3 posters and you are allocated about 12 villages* (IDI-KLN-HW-002)

*Again, those that cannot read will just pass by unless they decide to ask what the poster is about. What if they decide to not ask? He/she will not know* (IDI-KLN-OL-004)

*The IEC materials are usually in Swahili and CDD passes the messages in the local dialect. This means they translate them to their understanding causing delays in mobilization activities* (SSI_NTDC_01)

*The one day allocated for the training is not enough to cover the content. We focus mostly on how drugs are dispensed and reporting tools. We usually do not cover the content in the IEC but we ask the CDDS if they have issues during mobilization to inform us* (IDI-KYF-HW-003)

#### MDA Planning, implementation, and follow-up

Although the development of IEC materials was executed by the National NTD office, the county and sub-county officers recommended that if given the responsibility, they could help on messaging and development of IEC materials as they understand their communities better. The county NTD program depends on the national NTD program for support.

*The MDA activities are perceived as national government activities and therefore most planning and other related activities are done at the national level. The county and sub-county levels usually feel left out. We could be allowed to organize the MDA activity, messaging, and development of IEC materials that are appropriate to our communities* (SSI – CNTD_01)).

Both the county and sub-county coordinators as well as health workers interviewed acknowledged that supervision and follow-up of ACSM of MDA for LF were not sufficiently carried out due to low funding.

*We plan for MDA activities and even include them in the Annual Work Plans (AWP) for the county (Kilifi). Consequently, this is included in the budget. But funds are never allocated for the activity* (SSI_NTDC_01)

### Potential of stakeholders’ involvement and participation in resource mobilization for MDA delivery with a focus on ACSM activities

**Fig. 2:**
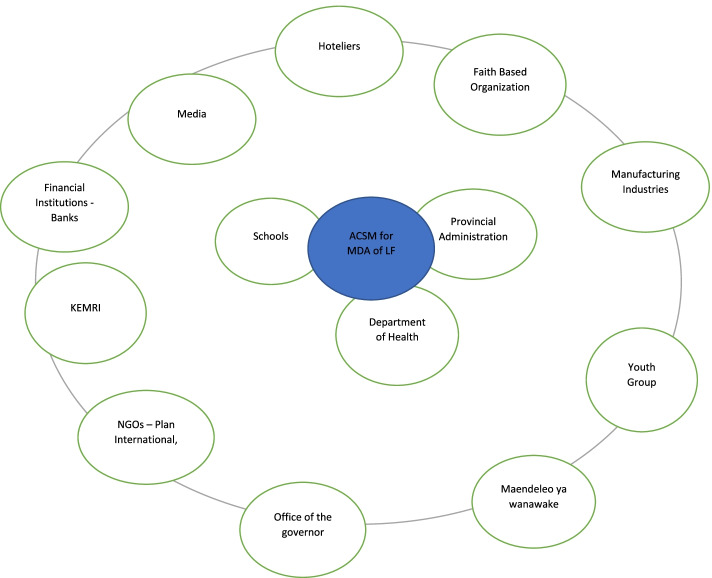
Example of Venn diagram showing the level of involvement in ACSM activities of MDA for LF at the county level. The big outer circle represents ACSM activities for MDA for LF. The middle of the outer circle is represented by a circle written ACSM activities. Circles close to the middle depict closeness to ACSM activities. The stakeholders that were identified as actively participating in ACSM activities are close to the middle while those that are far off from the middle were minimally engaged. The size of the circle depicts perceived importance of a stakeholder or partner in ACSM activities. All stakeholders and partners were identified as important. Their importance was based on expected roles in ACSM activities

The participants observed that Kilifi County had many stakeholders but most were never utilized or minimally involved to champion access of MDA medicines and ACSM to communities they serve. The meeting participants recognized and appreciated the importance of each stakeholder and partner, as shown in the size of the circle in Fig. 2. They also identified their level of involvement in ACSM as shown in Table 2. They agreed that apart from the identification of stakeholders, they needed to be actively involved in joint planning, directed messaging, communication, and sensitization to enhance uptake of medicines in the communities. Strengthening the role of stakeholder participation was therefore identified as crucial in resource mobilization thus improving awareness and information about MDA and LF. Below are some extracts from stakeholders’ meetings:

*You see there are many stakeholders and partners here. The problem is that we are not involved in this activity (referring to the MDA program). If we can be informed early, we can always support with whatever one has (Participant in the stakeholders’ meeting - Kilifi County)*

*There is a lot that the stakeholders and partners can participate in if they are involved early. We can chip in providing resources. Letters can be sent to us requesting assistance and I am sure most of us here will be willing to support the MDA with fuel, umbrellas, gumboots, airtime, and other things that officers and CDDs might require improving coverage of MDA (Stakeholders’ meeting – Kilifi)*

*In addition, we can participate in creating awareness to our congregations and informing them of the importance of taking the medicines (Religious leader – Representative- Stakeholders’ meeting - Kaloleni)*

However, it was noted that a few stakeholders including schools through the Ministry of Education, State Department of Social Protection, and State Department of InteriorServices and Co-ordination of National Government were involved mainly in social mobilization activities such as informing the residents about the dates of MDA activity.

*… the county has pledged support. We have had in*creased *advocacy and many sectors, departments and ministries have been involved. They include the State Department of Social Protection, in advocacy and Ministry of Education through schools (SSI-NTDC-01)*

**Table 2:** shows the stakeholders & partners identified and their level of involvement in ACSM activities

Stakeholders & partners identified	Level of involvement in ACSM activities
Government ministries - Ministry of education, department of health, Department of social services, Ministry of Interior,	High
Non-Governmental Organizations - Plan international, Afya Pwani, Population Services International	Low
Financial institutions: local banks;	Low
Local and community organizations e.g. Maendeleo ya wanawake, youth groups, women groups, religious and faith based organizations	Low
Media	Moderate
Research institution - KEMRI	Moderate
Manufacturing industries - Bamburi Cement, Mombasa Cement	Low
Hospitality industries – hotels	Low

### Preferred mode of awareness and ways to improve ACSM preparation, development, and dissemination processes

During the stakeholders meeting, FGDs and IDIs discussed the preferred modes of awareness and gave suggestions for the need to provide more information about the MDA and health education through a variety of channels for improved community mobilization and compliance as shown in Table 3. Stakeholders suggested channels such as the media through radio talk shows; public address systems and roadshows; focused meetings with women groups, youth groups, churches, and mosques. They also suggested that messages should be simple, easy to understand, and translated/disseminated in the local language to enable community members better to understand the importance of MDA for LF. Roadshows involve crisscrossing the whole village, announcing the MDA from a truck equipped with loudspeakers, and stopping where there were gatherings to distribute information brochures/leaflets and answer questions about the treatment.

*Health education is very important in this exercise. We have used focused meetings, roadshows and the media like Lulu FM in other programs and the reception has been very good.* (Stakeholder attendee - Kilifi County).

**Table 3:** Preferred mode of awareness and innovative ways improve ACSM preparation, development and dissemination processes

Preferred mode of awareness	Suggestions for improvement
**IEC materials – pamphlet, brochures**	- Prepare them in local language (Kigiriama)
	- Disseminate early to residents
	- Have pictures
**Banners**	- Place them in strategic places such as schools, churches, shopping centres
**CDDs**	- Train CDDs on messages to pass to household owners
	- Add time for awareness
**Barraza**	- Invite Health officials to give health education to residents
	- Facilitate organizers 0- chiefs, assistant chiefs and Barozis
	- with airtime
	- Attach other activities food, net distributions that bring residents together
	- Inform residents in good time
**Radio, TVs**	- Make announcements in the evening when he residents are indoors
	- Have talk shows and allow residents to ask questions
	- Use local radio and TV stations
**WhatsApp**	- MDA co-ordinators to provide simplified messages to be shared with WhatsApp groups so as to reach more people
**Roadshows and load speakers announcements**	- Vehicles with load speakers in community gatherings such as market or shopping centres
	- Use

#### Enhanced community meetings (barazas) with awareness attended by health officers

Community meetings, *Baraza,* were used to create awareness. These were called by community leaders such as the chiefs and assistant chiefs. In these meetings, community members were informed about MDA for LF exercise and encouraged to participate as well as inform others about the program. The community meetings were however stated not to be among the best awareness creation platforms because some community members absconded them for lack of incentives.

*Community members do not attend these meetings the way they attend when we are providing them with free food* (Stakeholder attendee - Kilifi County).

Importantly, the community meetings could be enhanced by the attendance of health officials to educate residents about MDA for LF including why it is important to take the drugs, demystifying myths and misconceptions concerning the disease and the drugs, side effects, and answering technical questions from residents.

*The Chief should call a meeting and the health workers are invited to create awareness in the meetings. In schools, we can have parents meeting and health officers can create awareness in such meetings too* (FGD-KLN-CM-AW-003 –P4)

*The Barazas are important and residents can be encouraged to attend when they hear there are health officials in attendance to give them more information about the disease and the drug distribution exercise (*IDI-KYF-OL-002)

*Let CDDs be accompanied by health workers so that they can explain the side effects to the community members (FGD-KLN-CM*-AW-003- P6)

Some participants especially the opinion leaders felt that door to door awareness creation strategy was still the best. This was preferred as it allowed a face to face communication with the household members. But sensitization about the MDA activity was only assigned 1 day during the implementation process. This, therefore, made it tedious, time-consuming and only a few households could be reached.

*.. door to door is the best strategy because if you schedule a meeting not all people shall come for the meeting and it allows a face to face interaction (*IDI-KLN-OL-004)

#### Mobile phones using WhatsApp and radio programs

Stakeholders suggested the need to use innovative awareness creation strategies and techniques to reach as many people as possible. They proposed the use of mobile phones to send messages about MDA for LF in areas with a local network through social media platforms such as WhatsApp groups. Additionally, they suggested that the use of local radio stations such as Kaya FM and Lulu FM would help inform people in the local language about the program, especially in rural areas. Once the community is made aware of the program, they could easily plan their activities and avoid missing the awareness teams. It was further suggested that engaging health officials knowledgeable with LF to discuss with community members on radio programs would also help create understanding and combat any negative beliefs and myths regarding the drugs and the disease.

*“. . .we could also use mobile phones like a WhatsApp group. Most of us have WhatsApp groups with many members from churches and mosques. If we could get simplified messages from the health officials, we could send them to our members for quick dissemination. [Stakeholders – chairman Faith-Based Organization representative).*

### Challenges with morbidity management and disability prevention services

Most of the stakeholders recounted the suffering that LF patients had to go through in their daily lives within the communities. They suggested that whilst MDA was meant for prevention, there was a need to hasten prevention services such as surgeries to those with chronic manifestations of hydrocele. It was reported that the MMDP program was started and identification of the patients was carried out. But, challenges were reported that hampered its effective implementation.

*… at the planning stage, about 550 operations were intended to have been done. Currently, over 100 are conducted. But, several challenges were encountered. Among them, functional theatre to execute the surgeries, wards to admit patients after surgeries, and personnel (Kilifi Stakeholders’ meeting attendee)*

Again, patients refuse to be registered when approached fearing surgical procedures saying that they are old and the procedure may lead them to early death.

## Discussion

The present study formed part of a larger study that aimed to address barriers of community participation and access to mass drug administration for lymphatic filariasis elimination in Coastal Kenya. The results of this study identified several challenges affecting the effective implementation of ACSM activities for MDA campaigns, potential for stakeholders’ participation in ACSM activities importantly highlighted the need for innovative strategies and techniques during preparation, development, and dissemination.

There were several challenges affecting social mobilization and awareness of MDA activities for LF. low involvement of stakeholders and partners, CDDs pressure to meet targets, the vastness of the area, limited time allocated for ACSM activities, and a few and inappropriate IEC materials were cited as major challenges affecting effective implementation of ACSM activities during MDA for LF. These challenges need to be addressed if the Kenyan program is to advance towards the elimination of LF. Kusi et., al 2020 also reported that low social mobilization and awareness for MDA lead to low knowledge of the transmission cycle contributing to misconceptions about the LF drugs increasing residents’ refusal to take drugs [13].

The current study observed that stakeholders were minimally utilized to champion MDA for LF activities in the county, sub-county, and community level as shown in Table 1 and Fig. 2.

However, stakeholders’ involvement was emphasized as an important factor in improving MDA implementation, especially in social mobilization and awareness creation activities. The stakeholders identified areas that they could be effectively involved in for improved ACSM activities for MDA. The following three key roles were identified: social mobilization and awareness, messaging and dissemination of information, and resource mobilization. Studies have shown that local partnerships are important in shaping participation and implementation as they provide a platform for building social capital, respectful relationships, engendering trust, and sustaining community support towards the MDA for LF program [15, 16]. A study by Silumbwe et.al 2019, reported that key stakeholders such as civic leaders promoted stakeholders’ buy-in, political goodwill and motivated the community members to participate in MDA for LF [17]. Importantly also, strategic international collaborations have been seen to equally contribute to facilitating participation in MDA for LF [18]. Stakeholders and partners in this study were willing to support the MDA for LF with resources such as gumboots, umbrellas, airtime, and fuel for officers’ moto bikes and fuel for vehicles used in the campaign. Resource mobilization from stakeholders and partners will be necessary as this will increase supervision, CDDs motivation, increase the households reached with messages on benefits of taking the drugs and their side effects which have been reported as barriers to improved MDA access [13, 19, 20]. Krentel et al., 2017 emphasize the importance of community engagement processes that promote participation being essential in achieving sustainable and successful implementation of MDA for LF [21]. Implementers have therefore to pay attention to such opportunities and start conversations that will lead to improved access.

The study also observed several challenges with IEC production, messaging, delivery, and dissemination. Specifically, the following barriers were identified: delays in delivery of IEC materials from the national NTD to the CDDs, packaging and messaging in English instead of –Kigiriama – the local dialect. The shortfall and delay of the delivery of IEC materials meant there was inadequate awareness creation to the community members. Again, the CDDs had to summarize and simplify the messages on their own by translating them to the local dialects. With the CDD level of education, the intended message might have been lost or distorted while summarizing and translating into the local dialects. Again, the 1 day allocated for awareness creation was too short to reach the assigned (500) households with MDA messages. An opinion leader who accompanied CDDs confessed that they hurriedly created the awareness and could not sufficiently answer to the issues that residents inquired about due to time constraints. Considering that most CDDs have a low level of education, program planners should ensure that the IEC materials are delivered early enough – a week before the MDA, are in the local dialect and that more time is allocated for social mobilization and awareness creation to allow the CDDs reach households with MDA information. Identifying barriers that persist across different health behaviours such as lack of time (due to family, household and occupational responsibilities), access issues (to transport and facilities), entrenched attitudes, restrictions in the physical environment and lack of knowledge can inform the design of tailored interventions for the community [22]. Intensified IEC activities, including the development and distribution of posters, flyers, leaflets, brochures, and radio and TV broadcasting of health messaging in the local language to promote awareness of drug distribution activities, provide health education, and promote behaviour change, to increase treatment coverage [23–25]. Enhanced ACSM activities of MDA for LF in Urban Kenya saw an increase in coverage in experimental clusters of 72.2% compared to 70.4% in control clusters with standard ACSM activities [24].

The current study recommended several measures to improve the efficiency and appropriateness of ACSM activities for MDA. They included allocation of sufficient time for awareness creation and health education, IEC to be produced in the local dialect, and utilization of innovative techniques such as WhatsApp when conducting ACSM activities. These recommendations are consistent with findings of a study conducted in another part of sub- Saharan Africa [23]. A study from Sierra Leone showed that the use of innovative and more “modern” sensitization techniques, enabled the reaching of individuals and institutions that had otherwise been unaware of MDA for LF [26]. The mandate of developing educational materials should be left with the local health officials and partners as they will consider their local experiences and other contextual issues of their communities. This needs to be encouraged and reinforced especially during this era of Corona Virus pandemic thus fostering community ownership [27, 28].

Concerning the limitations of the study, the purposive selection of participants might have resulted in selection bias. Secondly, social desirability bias may have affected the response of the participants. To minimize these limitations, different methods were used in data collection which allowed for triangulation by experienced social science researchers.

## Conclusions

Strengthening ACSM strategies and techniques is essential for improving residents’ awareness of the MDA of LF. As identified in this study, a deliberate effort is required in sensitizing residents about the risk and benefits of diethylcarbamazine (DEC). In addition, MDA for LF teams will need to ensure proper planning for ACSM activities including messaging, disseminating as well as ensuring training, and supportive supervision and monitoring for effective implementation of these activities. A social-ecological approach should be adopted where all sectors in the community including family, communities, institutions (local and national, government and non-governmental), and policymakers contribute to the design, development, and dissemination of target-specific, appropriate, responsive, and acceptable ACSM strategies and techniques. The findings of this study was used to improve ACSM activities in subsequent MDA campaigns for LF. The results were shared with respective stakeholders including MOH planners at all levels of National, Counties, and Sub – County, community drug distributor, partners, and the community for implementation.

## Supplementary Information

**Additional file 1 :** FGD Guide txt.

**Additional file 2 :** Minutes of stakeholders meeting in Kilifi txt.

**Additional file 3 :** minutes of stakeholders meeting in Kaloleni txt.

## Data Availability

The datasets and materials generated during the current study are available upon request from the Director-General, KEMRI through director@kemri.org

## Abbreviations

ACSM: Advocacy, Communication, Social and Mobilization
CFA: Circulating Filarial Antigen
DVB-NTD: Division of Vector-Borne and Neglected Tropical Diseases
CDD: Community drug distributor
CHEW: Community health extension worker
KEMRI: Kenya Medical Research Institute
LF: Lymphatic Filariasis
MDA: Mass Drug Administration
NTDs: Neglected tropical diseases
WHO: World Health Organization

## Footnotes

1
** refer to Kenyan constitution 2010.*

## References

[1] World Health Organization (2018). Global programme to eliminate lymphatic filariasis: progress report, 2017.

[2] Hotez PJ, Kamath A (2009). Neglected tropical diseases in sub-Saharan Africa: review of their prevalence, distribution, and disease burden. PLoS Negl Trop Dis.

[3] Bockarie MJ, Rebollo M (2016). Reducing the population requiring interventions against lymphatic filariasis in Africa. The Lancet. Glob Health.

[4] Gyapong J, Boatin B. Neglected tropical diseases-sub-Saharan Africa. New York Dordrecht London: Springer International Publishing; 2016.

[5] Ndeffo-Mbah ML, Galvani AP (2017). Global elimination of lymphatic filariasis. Lancet Infect Dis.

[6] Moraga P, Cano J, Baggaley RF, Gyapong JO, Njenga SM, Nikolay B (2015). Modelling the distribution and transmission intensity of lymphatic filariasis in sub-Saharan Africa prior to scaling up interventions: integrated use of geostatistical and mathematical modelling. Parasit Vectors.

[7] Njenga SM, Kanyi HM, Mutungi FM, Okoyo C, Matendechero HS, Pullan RL (2017). Assessment of lymphatic filariasis prior to re-starting mass drug administration campaigns in coastal Kenya. Parasit Vectors.

[8] Kenya National Bureau of Statistics (2009). Kenya population and housing census: analytical report on population projections.

[9] Ministry of Health (2019). The Kenya national breaking transmission strategy for soil-transmitted Helminthiasis, Schistosomiasis, Lymphatic Filariasis and Trachoma 2019-20233. Strategy.

[10] Silumbwe A, Zulu JM, Halwindi H, Jacobs C, Zgambo J, Dambe R (2017). A systematic review of factors that shape implementation of mass drug administration for lymphatic filariasis in sub-Saharan Africa. BMC Public Health.

[11] Dissak-Delon FN, Kamga G-R, Humblet PC, Robert A, Souopgui J, Kamgno J (2019). Barriers to the National onchocerciasis control programme at operational level in Cameroon: a qualitative assessment of stakeholders’ views. Parasit Vectors.

[12] Krentel A, Fischer PU, GJ. W. (2013). A review of factors that influence individual compliance with mass drug administration for elimination of lymphatic filariasis. PLoS Negl Trop Dis.

[13] Kusi C, Steinmann P, Merten S (2020). The fight against lymphatic filariasis: perceptions of community drug distributors during mass drug administration in coastal Kenya. Infect Dis Poverty.

[14] Jackson K, Bazeley P. Qualitative Data Analysis With NVivo. 3rd ed. Thousand Oaks: Sage Publications; 2019.

[15] Malina R (2010). Physical activityand health of youth.

[16] Liese B, Rosenberg M, Schratz A (2010). Progmmes, partnerships, and governance for elimination and control of neglected tropical diseases. Lancet.

[17] Silumbwe A, Halwindi H, Zulu JM (2019). How community engagement strategies shape participation in mass drug administration programmes for lymphatic filariasis: the case of Luangwa District, Zambia. PLoS Negl Trop Dis.

[18] Sodahlon YK, Dorkenoo AM, Morgah K, Nabiliou K, Agbo K, Miller R (2013). A success story: Togo is moving toward becoming the first Sub-Saharan African Nation to eliminate Lymphatic Filariasis through mass drug administration and Countrywide morbidity alleviation. PLoS Negl Trop Dis.

[19] Njomo DW, Kimani BW, Kibe LW, Okoyo C, Omondi WP, Sultani H (2020). Implementation challenges and opportunities for improved mass treatment uptake for lymphatic filariasis elimination: Perceptions and experiences of community drug distributors of coastal Kenya. PLoS Negl Trop Dis.

[20] Njomo DW, Kibe LW, Kimani BW, Okoyo C, Omondi WP, Sultani H (2020). Addressing barriers of community participation and access to mass drug administration for lymphatic filariasis elimination in Coastal Kenya using a participatory approach. PLoS Negl Trop Dis.

[21] Krentel A, Gyapong M, Mallya S, Boadu NY, Amuyunzu-Nyamongo M, Stephens M (2017). Review of the factors influencing the motivation of community drug distributors towards the control and elimination of neglected tropical diseases (NTDs). PLoS Negl Trop Dis.

[22] Kelly S, Martin S, Kuhn I, Cowan A, Brayne C, Lafortune L (2016). Barriers and facilitators to the uptake and maintenance of healthy behaviours by people at mid-life: a rapid systematic review. PLoS One.

[23] Dembélé M, Bamani S, Dembélé R, Traoré MO, Goita S, Traoré MN (2012). Implementing preventive chemotherapy through an integrated national neglected tropical disease control program in Mali. PLoS Negl Trop Dis.

[24] Njomo DW, Mukoko DA, Nyamongo NK, Karanja J (2014). Increasing coverage in mass drug administration for Lymphatic Filariasis elimination in an urban setting: a study of Malindi Town, Kenya. PLoS One.

[25] World Health Organization (2017). Child health and development: information, education, and communication.

[26] Hodges MH, Smith SJ, Fussum D, Koroma JB, Conteh A, Sonnie M (2010). High coverage of mass drug administration for lymphatic filariasis in rural and non-rural settings in the Western Area, Sierra Leone. Parasit Vectors.

[27] Krentel A, Gyapong M, McFarland DA, Ogundahunsi O, Titaley CR, Addiss DG (2021). Keeping communities at the centre of efforts to eliminate lymphatic filariasis: learning from the past to reach a future free of lymphatic filariasis. Int Health.

[28] World Health Organization and the United Nations Children’s Fund (UNICEF). Community-based health care, including outreach and campaigns, in the context of the COVID-19 pandemic. Geneva: WHO; 2020. https://www.who.int/publications-detail/community-based-health-care-including-outreach-and-campaigns-in-the-context-of-the-covid-19-pandemic.

