# Supplementary material for: Towards elimination of Lymphatic Filariasis in Kenya: improving advocacy, communication and social mobilization activities for mass drug administration, a qualitative study
Source: Trop Dis Travel Med Vaccines. 2022 Jun 6;8:16. doi: 10.1186/s40794-022-00172-8 (PMC9167906; doi:10.1186/s40794-022-00172-8)
Supplement: Supplementary file 1 — Additional file 1 : FGD Guide txt. [file 40794_2022_172_MOESM1_ESM.docx]

**Focus Group Discussion for Community Members (women, men and youth groups)**

| Instructions:   - This form should be used for FGDs for the community members. - If the participants refuse to answer a question, circle the number of the question and do not mark any answers for that question. - After obtaining informed consent, read the following instructions to the participants: |
| --- |
| **“I am going to ask you questions about the Filariasis control program, so as to collect information about your knowledge of LF and opinion of MDAs, your community member’s willingness to participate in the program and their preferences for being reached during the campaign. Please answer the questions as honestly as you can remember. Your information which will be tape recorded will be kept private and this form will not have your name anywhere. If you have any questions or do not understand what I am asking you at any time, please ask for clarification. Some questions may prove embarrassing to you.**  **Please remember that you do not have to answer any questions that you do not want to answer and you may discontinue the discussion at any time. Do you have any questions before we begin?”** |

**Socio-Demographic Characteristics**

| Sub-county: | | | Moderator: | | |
| --- | --- | --- | --- | --- | --- |
| Ward: | | | Note taker: | | |
| Date of FGD: | | | Time start: | | |
| Location of FGD: | | | Time stop: | | |
| Participants at start | | | Debrief notes | | |
| Participants at stop | | |  |  |  |
| **Participant** | **Age** | **Sex** | **Level of Ed.** | **Religion** | **Occupation** |
| 1 |  |  |  |  |  |
| 2 |  |  |  |  |  |
| 3 |  |  |  |  |  |
| 4 |  |  |  |  |  |
| 5 |  |  |  |  |  |
| 6 |  |  |  |  |  |
| 7 |  |  |  |  |  |
| 8 |  |  |  |  |  |
| 9 |  |  |  |  |  |
| 10 |  |  |  |  |  |

**Focus Group Questions**

1. What are the common diseases in your village? If swollen genitals (hydrocele) and swollen limbs (lymphoedema) are not mentioned, probe

What about swollen limbs?

What about swollen genitals?

2. What causes swollen limbs?

3. What causes swollen genitals?

4. Who are normally at risk of getting swollen limbs?

5. Who are normally at risk of getting swollen genitals?

6. How severe/dangerous can swollen limbs and genitals be?

7. Tell me about MDA for swollen limbs and swollen genitals in your community.

Probe about

1. Length of MDA
2. Mode of drug distribution and
3. Interaction with drug distributor.

8. How do people in your village feel about?

1. The length of MDA?

9. How do people in your village learn about the MDA?

1. (Probe about the sources of information and their sufficiency in informing the communities about MDA).
2. (Probe about the frequency of informing the communities about MDA).
3. (Probe about preferred ways of awareness creation during MDA campaigns)

10. Have you and your village members been taking the drugs during the MDAs?

11. How many times have you and your village members taken these drugs?

12. Do you know of people from your village that had problems (within 2 days)

after taking the drugs?

13. Did people from your village have problems with the drugs? (probe about size, number and taste of the drugs).

14. Would your village members be interested in taking the drugs next time they are given?

15. Next time the drugs are distributed would you want them distributed as in the last time?

If not, please tell me how you would prefer the drugs to be distributed. Probe about

Method of distribution

Distributors

Duration of distribution

**THANK YOU VERY MUCH FOR YOUR COOPERATION**
