# Supplementary material for: Towards elimination of Lymphatic Filariasis in Kenya: improving advocacy, communication and social mobilization activities for mass drug administration, a qualitative study
Source: Trop Dis Travel Med Vaccines. 2022 Jun 6;8:16. doi: 10.1186/s40794-022-00172-8 (PMC9167906; doi:10.1186/s40794-022-00172-8)
Supplement: Supplementary file 2 — Additional file 2 : Minutes of stakeholders meeting in Kilifi txt. [file 40794_2022_172_MOESM2_ESM.docx]

**LF- MDA research feedback meeting with stakeholders on held on 22^nd^ October 2018 at Kilifi County (Directors Board room)**

Present:

The meeting was attended by 28 members drawn from county health department, Education, KEMRI HQ, KEMRI- CGMRC, County Commissioners, Maendeleo ya Wanawake, World Vision, Media from Kilifi Governor’s office, Church Representation and National NTD programme as indicated in the below attendance list

The meeting started at 8. 30 a.m. with prayers from Patron Mwema. This was followed by a welcoming remarks by the County Director of Health Vincent Iduri. He later called upon participants to introduce themselves. After the introduction he called upon opening remarks from KEMRI and MoH-NTD Program.

After the opening remarks, Wyckliff Omondi provided the participants with an overview of NTD program in Kenya. He later narrowed down to LF program detailing the Kenya’s progress towards LF elimination.

After, Dr. Doris Njomo took the participants through research study to improve access to mass drug administration for Lymphatic Filariasis elimination using participatory approach among communities of conducted in Kaloleni Sub County in Kilifi County.

After the two presentations, the participants sought clarification the two areas covered and the following feedback ensued;

One of the participants wanted to know effectivity of previously skipped mass drug administration in phase one of MDA implementation.

1. In response from National NTD Official noted that even though the implementation was inconsistent with the elimination goals, subsequent survey in areas with intermittent MDA have shown reduced prevalence and as such may not be said to have been wasted.

Another member noted why there was no integration of LF activity with those of malaria given the fact that the two diseases are mosquito borne. Additionally the participant wanted to know if there is considerable change in biting behavior of mosquitoes that may have bearing towards LF transmission.

- It was noted that in as much as the two diseases are due to mosquito certain control aspects are being looked into such as sharing information on net distribution and usage. It was noted however that there are those mosquitoes that transmit LF and do not do the same for malaria.
- It was noted that the biting behavior of mosquitoes have not significantly changed to raise alarm of transmission and as such continued use of bed nets and other mosquito control will lead to sustained decline in transmission of LF.

A participant was curious to know why the current recommended regimen that would shorten the treatment period from 5 years to 2 years was not being used in Kilifi.

- It was noted that the triple therapy (IDA) was recommended was approved by WHO in 2017. As such it is to be implemented in areas that warranty the regimen. To this only Lamu East and West and Jomvu qualified based on the current IDA treatment criterion. Additionally, it is resource intensive and with limited funding may not be effectively implemented in Kilifi.

A member emphasized on the need to have a robust awareness of LF among the community members and suggested inclusion of women and key leaders in the program. This sentiment was echoed by one of the CHMT member who noted that in the past health officers were involved in awareness’ creation as opposed to current dispensation.

- It was noted that in the current implementation, health staff are key to awareness creation and as such both County, Sub county and CDD supervisors trained to implement MDA implementation need to champion the sensitization in respective areas they are charged. To this, all trained and engaged officer’s need to disseminate LF activities to one another using various platforms.
- It was noted that key MDA stakeholders vary from one place to another and as such each Sub County is tasked to assess its own relevant MDA stakeholders such as; administration, women, youth people with disability, political leadership, religious leaders and involve then accordingly. This mandate rest with the county and implementing Sub Counties etc.
- To this, the county team was urged to step up social mobilization using the funds provided by diversifying communication channels and embrace use of social media.
- It was recommended that through the office of the Governor, integration of the Balozis and local administrators to disseminate MDA information will be essential.

Office of the County commissioner sought to know the reason why some community members believe that the MDA medicines are aimed at capping life at 52 years. , infertility

- This was attributed to low awareness leading to continued misconception of LF and medicines. As such health promotion was recommended using key messages at different platforms.

A participant asked why intended surgical operations were low and if so was it by design

- It was noted that at the planning stage about 550 operations were intended to have been done. Currently over 100 has been conducted. To this it was noted that a number of challenges were encountered. Among them, functional theatre to execute the surgeries, Wards to attend to the patients and personnel. It was also noted that due to misdiagnosis, the high number reported quite a number are hernias.

One participant requested to know the mechanism that will be used to persuade some of the religions such as Imani Moja that advocate nonuse of medicines among faithful’s.

- It was stated that adequate health promotion and education among the identified members may lead to change of believe hence need to approach the faith based organization and educate them on the benefits of participating in MDA.

After the discussion Dr, Njomo proceeded to take the participants through the study Key findings of the first study phase. In her presentation, she noted the following key recommendations

- Need of Health promotion to enhance knowledge about LF the disease, drugs, MDA
- Need to increase awareness time
- House to house delivery method was most preferred.
- Need to administer MDA medicines early morning or late in the evening.
- Need for transparency during CDD Selection
- Necessity to provide supportive supervision to CDD
- Adequate provision of CDDs with enough MDA materials, medicines and data capture tools.

Need to improve the hygiene when dispensing the medicines by CDDs.

After the presentation, the following discussions ensued:

One participant sought to know why in the study finding the coverage was high 81% yet the sub county did not reach the 65% minimum coverage.

- It was rectified that in 2017 the sub county attained a coverage of 79% yet the study found out that among the interviewed participants 81% ingested medicines.
- It was furthers illustrated that the above coverage was representation of 2017 and not 2016 and 2015 low coverages.

One of the participant’s wanted to know if there are messages tailor-made towards side effects

- The participants were informed that side effects are expected but more due to DEC. It was also noted that those with parasites are likely to experience stronger effects since the medicine are acting on microfilariae’s however if the effects persist they should seek attention in the nearest health facility.

One participant asked why the CDDs are provided with few hours of training yet they are tasked of passing the information to the community members.

- In response, County NTD coordinator informed the participant’s CDD training is for a full day. To ensure utilization of the day, CHEWs need to seek venue near the areas where CDDs come from to reduce time spent to the training venue. Additionally due to limited funding and logistical issues training are timed few days before MDA to allow CDDs take with them the MDA commodities.
- It was also emphasized that Training and CDD selection must be within the criteria provided; must be from the community where they treat and able to read and write.
- In fact it was noted that some areas, the trainings are split. In vast areas training are conducted separately. There is facilitation for these trainings
- It was also observed that awareness need to be conducted continuously and not as one off activity during the MDA

A member suggested that there is need to move from health education to health promotion. FGD is better avenue. Leaders at the county, sub-county level,

After discussion of preliminary results Dr. Kibe took the participants through the role of stake holders by engaging the participants through plenary discussion.

Each of the participants was requested to provide at least two key MDA stake holders that are relevant for successful implementation of MD in Kilifi County. The provided list of stakeholders were written on flip chart.

Later, the participants were challenged to rate the contribution of the listed participants during 2017 MDA. At the end of the exercise it was observed that most of the stakeholders were never utilized or minimally used to champion access of MDA medicines to communities they serve.

It was agreed that a part from identification of stake holders, joint planning, directed messaging, communication and sensitization needs to be embraced by all stakeholders to enhance uptake of medicines in the Sub County. Strengthening the role of stakeholder participation was therefore identified as crucial in improving the coverage of MDA

At the end of the meeting, Dr. Charles Mwandawiro facilitated the anticipated way forward on improving LF-MDA in Kilifi County. In his presentation, he urged the participants to ensure that the following six points are considered in order to administer successful MDA

1. Understand the problem by taking cognizance of the presence of LF diseases among the community members hence transmission,

2. Accept the fact that transmission can only be interrupted by taking medicines. Explore possibilities of health promotion to community members and educate them on why they are being provided with medicines, side effects and how to control and prevent LF.

3. How to Access to MDA medicines. He informed the team that the medicines are free and should be cautious on how they pass the benefits of the medicines to the community members to dispel myths that may surround them

4. Provision of adequate training of selected CHEWs and CDDs within the Sub County. The essence id to enable the team to explain any question that may arise among the community members while administering medicines.

5. Strategize on how to maximize on the house to house delivery of the medicines. Ensure that missed persons are revisited to maximize coverage.

6. Lastly, advocacy, community sensitization and mobilization to be given adequate time to ensure that MDA information is passed to the community areas.

Closing remarks was provided by County Director of Health Kilifi, Administration office and final vote of thanks by the County NTD coordinator. Closing prayer was presided by the Maendeleo ya Wanawake Kilifi representative. The meeting was adjourned at 3.10 pm

**LYMPHATIC FILARIASIS WORKSHOP**

**STAKEHOLDERS’ SENSITIZATION AND PLANNING FOR 2018 MDA**

**22^nd^ October 2018**

**Venue: Director’s Boardroom**

**Master of Ceremony: Mr. Patrick Makazi**

**Rapporteurs: Dr. Lydiah Kibe and Mr. Wyckliff Omondi**

**KEMRI RESEARCH STUDY- RESULTS OF THE PRE-TEST PHASE**

Improving Access to Mass Drug Administration for Lymphatic Filariasis Elimination using a Participatory Approach among Communities of Coastal Kenya

| **TIME** | **ACTIVITY** | **FACILITATOR** |
| --- | --- | --- |
| 9.00 - 9.30 | Arrival and Registration | **Wyckliff Omondi-** National LF Program Focal Person |
| 9.30 – 9.40 | Welcome and Introductions | **Mr. Patrick Makazi-**NTD Coordinator, Kilifi County |
| 9.40 – 9.50 | Opening remarks | **Mr. Iduri – County** Director Department of Health, Kilifi and to call  **Dr. Anisa Omar** -CEC to give her remarks |
| 9.50-10.20 | Overview of NTD programme in Kenya- specific to LF Program | **Dr. Sultani Matendechero–**NTD National Coordinator |
| 10.20 – 10.40 | Overview of research study -Improving Access to Mass Drug Administration for Lymphatic Filariasis Elimination using a Participatory Approach among Communities of Coastal Kenya | **Dr. Doris Njomo** – Principal Investigator, KEMRI- ESACIPAC |
| 10.40-11.00 | ***Tea Break*** |  |
| 11.00-11.30 | Key Findings of the Pre-test Phase | **Doris Njomo** – Principal Investigator, KEMRI- ESACIPAC |
| 11.30- 12.00 | Role of stakeholders in LF-MDA | **Dr. Lydiah Kibe-** KEMRI -CGMRC |
| 12.00 – 12.30 | Plenary | **Dr. Lydiah Kibe,** KEMRI-CGMRC |
| 12.30 – 1.00 | Plenary Reflections | **Dr. Doris Njomo** – Principal Investigator, KEMRI- ESACIPAC |
| 1.00 – 2.00 | ***Lunch Break*** |  |
| 2.00 – 2.30 | Way forward on improving LF- MDA in Kaloleni/ Kilifi County | **Dr. Charles Mwandawiro** –Assistant Director, KCE,KEMRI- |
| 2.30 - 2.40 | Remarks by Government Representative | **Mr. Josphat Mutisya-** Deputy County Commissioner, Kilifi |
| 2.40 – 2.50 | Closing Remarks | **Mr. Iduri-** County Director, Department of Health |
| 2.50 - 3.00 | Vote of thanks & Announcements, Prayers | **Mr. Patrick Makazi-**NTD Coordinator, Kilifi County |
