# Supplementary material for: Towards elimination of Lymphatic Filariasis in Kenya: improving advocacy, communication and social mobilization activities for mass drug administration, a qualitative study
Source: Trop Dis Travel Med Vaccines. 2022 Jun 6;8:16. doi: 10.1186/s40794-022-00172-8 (PMC9167906; doi:10.1186/s40794-022-00172-8)
Supplement: Supplementary file 3 — Additional file 3 : minutes of stakeholders meeting in Kaloleni txt. [file 40794_2022_172_MOESM3_ESM.docx]

**Improving Access to LF MDA study feedback meeting with stakeholders on held on 23^nd^ October 2018 at Kaloleni sub- County in Deputy County Commissioners Board room**

**Present**:

More than 28 participants drawn key MDA delivery from health, administration, religious sector, non-state actors and community representative attended the meeting. (**Attached- List of Participants and Meeting Agenda)**

The meeting started at 10. 35 with prayers Imam Alii Athuman. This was followed by opening remark from Kaloleni SC-MOH Dr. Umi O. Bunu. She informed the meeting that the SC team aims at attaining above 80% coverage in the coming MDA. Additionally she informed the team of the CEC Kilifi support on 2018 MDA implementation.

After the opening remarks, Wyckliff Omondi enlightened the participants with an overview of NTD program in Kenya. He later narrowed down to LF program detailing the Kenya’s progress towards LF elimination.

Dr. Doris Njomo followed on, providing the team with an overview of research study to improve access to mass drug administration for Lymphatic Filariasis elimination using participatory approach among communities of Coastal Kenya.

After the two presentation, the participants sought clarification the two areas covered and the following feedback ensued;

One of the PHO from Kayafungo ward enquired on the impending hydrocele surgeries. It was noted that since the identification of the patients no surgeries had been conducted leading to impatience among the community members.

- In response by Mr. Patrick Makazi acknowledged funding challenge towards MMDP. He however noted that through the NTD program, two funding streams; MAP and END Fund were made available. To, this he clarified that three facilities were identified for the activity; Malindi, Kilifi and Mariakani. Out of the 3 facilities, surgeries were only actualized in Malindi and Kilifi since Mariakani facility could not manage the patients.
- The above information was echoed by the SC-MOH: who informed the meeting that they have been able to fix underlying issues at Mariakani facility and looks towards starting hydrocelectomies before the beginning of the 2018 MDA.
- One of the participants urged the county to equally step up the hydrocelectomy activities and assist the deserving patients.

Another participant who participated in community treatment requested the team to look into the number of treatment days from 3-5 so as to increases coverage.

- It was noted that towards the restart of the MDA in 2015 & 2016 the 5 day treatment period was portioned into: 1 day for registration, 3 treatment days and 1 mop up day. In 2017 it was noted that 5 treatment days was provided. Additionally it was noted that the rushed 2017 MDA activities was as result of prolonged electioneering campaign that impacted number of treatment timelines

One of the Chiefs asked on how to deal with the side effect of MDA medicines as some members complained after participating in the MDA.

- It was noted that all medicines have side effects. The participants were informed that the fact that DEC causes dizziness is well known hence the need to sensitize the residents on this and what they need to do should the events persist.
- It was also noted that if any of the community members experience adverse events after taking MDA medicines, they should be advised to immediately seek attention in the nearby health facility

Another administrator from Kinagoni sought to know why households in Kinagoni were not interviewed despite the fact that they were within the survey area.

- The participants were informed that even though the wards were the study areas, a number of villages were purposively selected based on their low coverages and as such only six villages were sampled out of the many that existed in the two wards.

After the discussion Dr, Njomo proceeded to take the participants through the study Key findings of the first study phase. In her presentation, she noted the following key recommendations

- Need of Health promotion to enhance knowledge about LF the disease, drugs, MDA
- Need to increase awareness time
- House to house delivery method was most preferred.
- Need to administer MDA medicines early morning or late in the evening.
- Need for transparency during CDD Selection
- Necessity to provide supportive supervision to CDD
- Adequate provision of CDDs with enough MDA materials, medicines and data capture tools.
- Need to improve the hygiene when dispensing the medicines by CDDs.

After the presentation, the following discussions ensued:

SC- MOH acknowledged some of the observed gaps during LF MDA implementation and assured the team that mitigation measures are being put in place. She proposed the need to incorporate nurses.

One of the participants informed the meeting that due to low number of health workers in the area, it would be difficult to deploy them to effectively mobilize communities.

The nursing officer supported the study findings and indicated that planning gaps have been observed in most of the implementation levels. She also reiterated that despite of the continued population growth, the program kept decreasing the number of CDDs and pay hence contributing to low coverages thus unable to cover allocated areas

SC- NTD coordinator echoed the findings of the preliminary results and noted that in advent of lo facilitation no good outcome would be expected. She noted that only chiefs and village elders were mandated to sensitize the community leaving out health workers. She noted that even though CDD numbers were increased no Supervisor was added. Lastly she noted that while the implementation was for 5 days, supervision was only allocated 2 days.

- The National LF coordinator reiterated that there has never been downward revision of number of CDDs provided. He illustrated that with a projected annual population increase at 3%, the number of CDDs and CHEW supervisors were equally expected to increase and that is the overall guide in allocating CDDs and CHEWs. He also clarified that since 2015, CDD payment had been 500 for 5 days contrary to 300/= highlighted by one of the participants.
- Additionally he elaborated that it is the normal expectation for a CDD to treat 500 people at the end of 5 treatment window period. Reorganization and target allocation CDDs is the mandate of Sub County.
- It was also highlighted that a CHEW need to supervise a maximum of 20 CDDs and support for supervision is for 3 days. Payment break down was provided; a total of 1,300 (500=lunch allowance, 500=transport,300 airtime)
- It was highlighted that both CHEW and CDDs should be drawn from nearby community areas where they serve. This was envisaged to minimize extra cost and enhance coverage in areas served.
- It was noted that CDD selection is mandated to CHEW and subsequent training of the CDDs. To this it was noted that each CHEW conducts his/her own training and this trainings should be close to areas where the CDDs are drawn to maximize the provision of one day currently provided.

Later in the afternoon, Dr. Kibe took the participants through the role of stake holders using participatory approach.

In the initial stages, each of the participants was requested to provide at least two key MDA stake holders that are relevant for successful implementation of MD in Kilifi county. The provided list of stakeholders was written on flip chart.

After identifying the MDA stakeholders, the participants were challenged to rate their contribution towards 2017 MDA. At the end of the exercise it was observed that most of the stakeholders were never utilized or minimally used to champion access of MDA medicines to communities they serve.

It was agreed that a part from identification of stake holders, joint planning, directed messaging, communication and sensitization needs to be embraced by all stakeholders to enhance uptake of medicines in the Sub County

At the end of the meeting, Dr Charles Mwandawiro facilitated the anticipated way forward on improving LF-MDA in Kaloleni / Kilifi County. In his presentation, he urged the participants to ensure that the following six points are considered in order to administer successful MDA

1. Understand the problem by taking cognizance of the presence of LF diseases among the community members hence transmission,

2. Accept the fact that transmission can only be interrupted by taking medicines. Explore possibilities of health promotion to community members and educate them on the medicines, side effects and how to control and prevent LF.

3. How to Access to MDA medicines. He informed the team that the medicines are free and should be cautious on how they pass the benefits of the medicines to the community members to dispel myths that may surround them

4. Training of CHEWs and ultimately CDDs key. The essence id to enable the team to explain any question that may arise among the community members while administering medicines.

5. Strategize on how to maximize on the house to house delivery of the medicines. Ensure that missed persons are revisited to maximize coverage.

6. Lastly, advocacy, community sensitization and mobilization to be given adequate time to ensure that MDA information is passed to the community areas.

Closing remarks was given by the Deputy County Commissioner Kaloleni. In his remarks, he thanked the participants for prioritizing their time to attend the meeting with aim of improving livelihood of members they serve. He reiterated that misconception of LF is still rive among community members due to low level of academics in the area. He noted that this has impacted the area adversely towards development agenda. He restated the support of administration to 2018 MDA call as contribution as a tribute to the presidential 4 key agenda on Health.

The meeting was adjourned at 4.30pm

**LYMPHATIC FILARIASIS WORKSHOP**

**STAKEHOLDERS’ SENSITIZATION AND PLANNING FOR 2018 MDA**

**23r^d^ October 2018**

**Venue: DCC’s Boardroom, Kaloleni Sub-county**

| **TIME** | **ACTIVITY** | **FACILITATOR** |
| --- | --- | --- |
| 9.00-9.30 | Arrival and Registration | **Wyckliff Omondi-** National LF Program Focal Person |
| 9.30 – 9.40 | Welcome and Introductions | **Mr. Patrick Makazi-**NTD Focal Person, Kilifi County |
| 9.40 – 9.-50 | Opening remarks | **Dr. Bunu -** Sub-County Director Health, Kaloleni |
| 9.50 – 10.20 | Overview of NTD programme in Kenya- specific to LF Program | **Mr. Wyckliff Omondi**–National LF Program Focal Person |
| 10.20 – 10.40 | Overview of research study -Improving Access to Mass Drug Administration for Lymphatic Filariasis Elimination using a Participatory Approach among Communities of Coastal Kenya | **Dr. Doris Njomo** – Principal Investigator KEMRI- ESACIPAC |
| 10.40 – 11.00 | ***Tea Break*** |  |
| 11.00 – 11.30 | Key Findings of the Pre-test Phase | **Doris Njomo** – Principal Investigator, KEMRI- ESACIPAC |
| 11.30-12.00 | Role of stakeholders in LF-MDA | **Dr. Lydiah Kibe,** KEMRI-CGMRC |
| 12.00 – 12.30 | Plenary | **Dr. Lydiah Kibe-** KEMRI -CGMRC |
| 12.30-1.00 | Plenary Reflections | **Dr. Doris Njomo** – Principal Investigator, KEMRI- ESACIPAC |
| 1.00 – 2.00 | ***Lunch Break*** |  |
| 2.00 – 2.30 | Way forward on improving LF- MDA in Kaloleni/ Kilifi County | **Dr. Charles Mwandawiro** –Assistant Director, KCE, KEMRI- |
| 2.30 - 2.40 | Remarks by Government Representative | **Mr. Paul Rotich Deputy-** Deputy County Commissioner |
| 2.40 – 2.50 | Closing Remarks | **Dr. Bunu-** Sub**-**County Director of Health |
| 2.50-3.00 | Vote of thanks & Announcements, Prayers | **Mr. Patrick Makazi- -**NTD Focal Person, Kilifi County |
